# Evaluation of United Kingdom (UK)—Windsor Framework and Comparison Against European Union (EU) Regulations for Medicines Regulation

**DOI:** 10.1007/s43441-025-00753-7

**Published:** 2025-02-12

**Authors:** R. B. Ankitha, Shailee Dewan, Francis Fernandes, Sharad Verma, Gowri M. Bhat, Pradeep M. Muragundi

**Affiliations:** 1 Department of Pharmaceutical Regulatory Affairs and Management, Manipal College of Pharmaceutical Sciences, Manipal Academy of Higher Education, Manipal, Karnataka 576104 India; 2 Deem Eligible Compliance Monitor for UK MHRA, 10, Davy Apartment, 229 Harrow View, Harrow, London Borough of Harrow, HA14GR UK; 3 Bioplus Life Sciences Pvt. Ltd., Pharmed Gardens, Whitefield Road, Bengaluru, Karnataka 560048 India

**Keywords:** Northern Ireland (NI), Great Britain (GB), MHRA, EMA, Falsified Medicines Directive (FMD)

## Abstract

The United Kingdom (UK)’s regulatory profile is changing following the UK’s exit from the European Union (EU). As a consequence, the Medicines and Healthcare products Regulatory Agency (MHRA) became more independent. Since then, numerous attempts have been made to ease the separation of the UK from the European Union, focusing mainly on Northern Ireland (NI), which is part of the UK but shares a land border with the EU. The Windsor Framework facilitates the relationship between the EU and the UK, including the role of the European Medicines Agency (EMA) and MHRA in NI. The review throws light on the implementation of the Windsor Framework detailing the key aspects, and the pre- and post-implementation changes in Northern Ireland, Great Britain and the Republic of Ireland. The Framework is useful for industries such as pharmaceuticals where regulatory approval and an uninterrupted supply chain are critical. Evaluating the Framework illuminate’s areas for improvement, threats, and scope for cooperation between the UK and EU authorities. The review details efficiency, costs, and market accessibility of medicines, to give a better representation of the regulatory position in NI. The study reveals the pros and cons of the Framework, to assist stakeholder evaluation of Marketing Authorisation Holders (MAHs) that have registered both in UK and EU markets.

## Introduction

A new set of regulations was drafted by UK and EU, on February 27th, 2023, ensuring a smoother stream of goods within the internal market of the UK and protecting the place of Northern Ireland (NI) in the Union [1]. This was introduced seeing the problems faced due to the disrupted supplies on account of delayed and partial implementation of the Ireland/Northern Ireland Protocol which was agreed upon by both EU and the UK post-Brexit [2]. After talks and engagement with stakeholders, the EU and UK collaborated to identify a solution to such practical challenges embodied in the agreement of the Windsor Framework, a political and legal contract that addresses the pragmatic difficulties faced by businesses in, and supplying, Northern Ireland and Ireland due to Brexit [3].

The Windsor Framework also addresses the practical deficit introduced by the Northern Ireland Protocol. As per the previous Protocol, medicines for Northern Ireland had to meet the requirements set out by both EU and UK regulatory systems, which caused several issues for the companies that supplied the medicines. This study elaborates further on these issues. The Windsor Framework removes barriers between Great Britain and Northern Ireland that were formally required (but never legally introduced) regarding their regulations for access to medicines [4].

The Windsor Framework establishes a new regulatory settlement for product licensing, labelling, and the Falsified Medicines Directive (FMD) [5]. The Framework ensures that a rigid border is avoided between the Republic of Ireland and Northern Ireland following the UK’s exit from the EU. [6].

A UK-wide licensing option is provided to Marketing Authorisation Holders (MAHs) to commercialise their products throughout the UK (including NI) while maintaining a single license and the same packaging for the whole UK. As part of the NI protocol, Novel medicines such as medicines for cancer required the EU Commission’s approval for marketing authorisation in NI but this was not enforced effectively. However, with the Windsor Framework in place, the MHRA will be responsible for the UK-wide licensing of such medicines [7].

The Windsor Framework was introduced following the unprecedented challenge of COVID-19 which demonstrated the difficulty of maintaining a continuous flow of medicinal products into Northern Ireland due to nontransparent, ineffective, and potentially dangerous contracts [8]. In the market of NI, EU laws govern the research, development, manufacturing control, and supply/procurement of pharmaceutical products. Pre-Brexit, the UK was dependent on the EMA for its policies and regulations for novel medicines. The MHRA is now responsible for designing its regulations and requirements for the whole of the UK [8, 9].

This review will also depict the path that MAHs can choose after implementation of the Windsor Framework. It will focus on identifying differences in medicinal product approvals, other requirements for compliance and post-marketing surveillance between the UK and EU.

## Current Regulatory Landscape in Northern Ireland

As part of the Ireland/ Northern Ireland Protocol, NI though a part of the UK, was majorly governed by the EU regulations until 31 December 2024. Though the Northern Ireland protocol was not fully enforced, activities like the movement of medicines into Northern Ireland required fulfilment as per Annexure 2 of this Protocol (i.e., mutual assistance between EMA and administrative authorities in each member state to ensure the correct application of laws/directives during custom flow), along with the application of EU rules on importation and unique identifier requirements referring to the Directive (EU) 2022/642 [10].

A qualitative comparative analysis of UK/EU regulations detailed in this narrative review provides an evaluation of the requirement of the UK Windsor framework over the EU regulations partially applied as per the Ireland/ Northern Ireland protocol in NI.

A few of the areas of Ireland/Northern Ireland protocol were implemented. Implementation and its shortcomings are comparatively evaluated below.

### Comparative Evaluation of EMA-MHRA Regulation and its Impact on the Current Scenario in Northern Ireland After the Implementation of the Windsor Framework

#### Existing Licensing Process

The medicines that need to be supplied to NI currently require authorisation by both EMA and/or MHRA.

- EMA: Within the EU, a human medicinal product can be registered by the European Commission using its centralized process or by the member states (included Northern Ireland as its member state until 31 December 2024) and their respective national competent authorities using a decentralized, nationalized or mutual recognition process [11].
- MHRA: Medicines that were outside the scope of the EU centralised procedure could also be approved by MHRA for use and supplied to NI through Northern Ireland MHRA Authorised Route (NIMAR) or exclusively licensed as Product Licensed in Northern Ireland (PLNI) [12].

Thus, a single medicinal product might possess two Marketing Authorisations (MAs), one issued by the MHRA and another by the EMA.

#### Qualified Personnel (QP) Release

Until 31 December 2024, NI was considered a member state of EU and the QP release of batches (i.e., certification of the finished product by personnel appointed by the manufacturer or importer as described in the Marketing authorisation of the product) was mandatory if the product is originated from any other country outside EEA [13].

- EMA: Any batch that enters the EU (including NI) has to be verified by a QP located in EU.
- MHRA: Post-Brexit, MHRA was not responsible for the QP release of batches in Northern Ireland [13]. However, medicines such as Product Licensed in Great Britain (PLGB) if QP-certified were supplied to NI through the NIMAR [14].

#### Unique Identifiers on Packs (Falsified Medicines Directive—FMD)

- EMA: As part of the serialization process EU Delegated Regulation 2016/161, any medicine to be authorised under the EMA requires unique identifiers (i.e. inclusion of barcodes) and tamper-evident on the packs to be sold in the EU.
- MHRA: The packs being supplied into the UK do not mandatorily require the unique identifiers. However, MAHs are encouraged to retain the tamper-evident packs. However, products shipped to NI via GB using the Northern Ireland MHRA Approved Route (NIMAR), usually did not possess any FMD features as it is not a UK regulatory requirement and was the responsibility of the MAHs to ensure the authenticity of the products [12, 15].

A single product thus if possessed MA’s issued by both MHRA and EMA, the absence of unique identifiers led to the assumption that UK medicines are counterfeits of EU version.

#### Authorisation of Innovative Products Based on Their Priority

- EMA: A specialised route is established by EMA for the Authorisation of Innovative Medicinal Products based on their priority—PRIME (Priority Medicines). This is mainly evaluated based on the major therapeutic action or demonstrated potential critical meaningful impact by the novel medicines. The earliest entry point for application is during the exploratory clinical phase of the medicines [16].
- MHRA: On the other hand, Innovative Licensing and Access Pathway (ILAP), provides an opportunity for new medicines that target Rare Diseases/which helps in significant improvement of Public Health. The earliest entry point for application is during non-clinical development of medicine [17].

A novel medicinal product awaiting registration in NI had differing registration periods in MHRA (approximately 12–14 weeks) and EMA (40 Days). Hence, a medicine available in the Republic of Ireland might not be accessible in Northern Ireland due to the involvement of either regulatory authority [16, 17].

#### Advertising and Promotion of Products

- EMA: The advertising and promotion of any Human medicine product is done as per the Directive 2001/83/EC (Article 97). EMA itself includes numerous member states and National regulatory agencies that suggest the guidelines as per their local requirements. Hence, it is usually preferred to follow the regulations as per the regions where a product is intended to be marketed. It is up to the MAHs to establish their regulation as per their market involvement [18].
- MHRA: MHRA establishes a clear guideline, the Blue Guide, which is a detailed descriptive set of rules to be followed for any medicine to be promoted in the UK [19].

Thus the public in the Northern Ireland had exposure to varied advertising and promotional exposure for the same product based on the source of importation.

#### Supply Route

- EMA: The EU regulations until 31 December, 2024 were primarily applicable to the supply of medicines across the NI. In case, the Qualified Personnel (QP) certification (and batch testing) is done in Great Britain, medicines with a valid marketing authorisation of EMA could be supplied to Northern Ireland. If QP certification (and batch testing) is done in the EU/EEA, medicines could also be supplied to Northern Ireland via Great Britain, without any additional importation control [12].
- MHRA: Products with MAs obtained from the MHRA were classified into the following 2 categories:

- Product Licensed in Northern Ireland (PLNIs): Products with MAs in NI and manufacturers or wholesalers from EEA, are required to follow Falsified Medicines Directive (FMD) mandatorily.
- Product Licensed in Great Britain (PLGBs): For supply of PLGBs to NI, they required approval by the Department of Health and Social Care (DHSC) and the MHRA via NIMAR (Northern Ireland MHRA Authorised Route) as they did not possess FMD.

Thus, there exist multiple packs of the same product being supplied through various routes into Northern Ireland, leading to ambiguity among the general public. Also, due to excessive documentation and import checks on the goods coming into NI, there was no steady supply chain for a single type of product [12].

#### Pharmacovigilance

NI was monitored by both EU and UK for PV requirements.

- EMA: Pharmacovigilance is carried out in EMA by the EudraVigilance system. The ICSRs (Individual Case Safety Reports) and PSURs (Periodic Safety Update Reports) records with MAs in Northern Ireland had to be mandatorily submitted to EudraVigilance. For the products with MAs in the UK, Authorised Medicinal Products (AMP) records could be submitted voluntarily based on their requirement, but users had to mention ‘United Kingdom (GB)’ as the referenced country under the category of ‘Non-EU authorisation procedure’ [20].
- MHRA: For products with parallel import permission (both UK and EU markets) or medicines named for patient use or nationally authorised medicines in UK by EMA before 2021, MHRA had readable access as products were authorised and available in Northern Ireland only. This is recorded in Article 57 also called as EudraVigilance Database [20].

Thus, for all products accessible to public in Northern Ireland, Pharmacovigilance was monitored by EMA, and read-only access was provided to MHRA based on the severity of the case.

## Windsor Framework Implementation and the Future of Northern Ireland Market

The Windsor Framework introduced new regulations in the UK from 01 January 2025, specifically for the above situation in Northern Ireland for product licensing, labelling, and supply. The MHRA can approve and license medicines in the entire UK (England, Scotland, Wales and NI) and ensure that the same packs of medicines can be supplied throughout. The disapplication of the EU FMD safety features for medicinal products that are authorized and commercialized in NI—as was already the case in Great Britain—is also highlighted [21].

### Key Provisions of Windsor Framework

The implementation of Windsor Framework is governed by its numerous provisions which can aid a stakeholder in adapting to the new regulatory requirements. The action for stakeholders is as below:

#### Licensing

- The MHRA authorises all new formulations and medicines that were regulated (Pre-Brexit) under the EU Centrally Authorised Procedure (CAP) for the UK market which are in line with the current definitions of medicines available in UK law.
- Category 1: Products such as biotech-derived products, Advanced Therapy Medicinal Products (ATMPs), Orphan Medicines, and New Active Substances are authorised by MHRA based solely on UK law.
- Category 2: Products such as Generics, hybrids, biosimilars and products not included in centralised procedure are authorised by MHRA based on either the UK or EU law as applicable.
- All PLGBs will be converted into UK-wide licenses and if any MAs hold both PLGB and PLNI, they must withdraw the PLNI immediately.
- Northern Ireland Licensing: A NI-only license can be used for UK-wide applications if the MAH is based in NI, called the Unfettered Access Procedure [22].
- Parallel Import Licenses (PLPI): Some MAHs who marketed their products in both GB and Europe, possessed Parallel Import Licenses, which allowed MAHs to distribute in the GB while maintaining the same inventory for GB and EU (including NI). These packs will have to possess a ‘UK Only’ statement with a separate inventory for the UK and cannot re-enter the EU market. The GB-only PLPIs will also be changed to UK-wide and distributed including in Northern Ireland [23].

#### QP Batch Release of Licensed Products

QP personnel must be UK residents, (including in NI) for UK-wide distribution of products. If the QP is based in the EU, then a Responsible Person for Import has to certify that the FMD features on the packs are not registered in EU repositories. Assurance of ‘UK Only’ stickering on all packs entering the UK, effective immediately in GB is mandatory [14].

#### Authorisation and QP Release of Investigational Medicinal Products

Historically, the markets of Cyprus, Ireland, Malta and Northern Ireland have been dependent on Great Britain for medicinal product supply. The operators of these markets require the manufacturers to have, permanently and continuously, at least one qualified person in the EU or Northern Ireland. The additional burden is now resolved concerning Northern Ireland with the Windsor framework implementation, as all packs available in Great Britain can have easier access to Northern Ireland with the same Qualified personnel for both GB and NI [24].

#### Paradigm Shift in Northern Ireland

The framework includes a mechanism known as the “Stormont brake” that would enable the NI assembly to temporarily prevent any EU regulations from being imposed in NI, if it appears that the practices are too different and such regulations are hindering NI’s independence from the EU. The Windsor Framework procedure is complicated in nature. In practice, cooperation between regulatory authorities is a more favourable route to ease the travel of goods into Northern Ireland, which in turn will improve the relations between MHRA and EMA [25].

#### Protection of the EU’s Single Market

The Windsor Framework has also been designed to protect the integrity of the EU’s single market, to which NI will have unique access. The markets of Cyprus, Malta and Ireland are greatly dependent on Great Britain via Northern Ireland. The changing regulation in NI if unmonitored will affect the supply of products that are being supplied via NI. Hence, post implementation of Windsor Framework, products that are entering the EU via NI from Great Britain/other countries will also be scrutinized in customs unless specified as ‘not at risk’ by the EU Joint Committee. Similarly, the Windsor Framework’s complete application and implementation in NI is solely the responsibility of UK authorities acting in NI and not the responsibility of its neighboring European states. This preserves the integrity of the EU’s single market for goods, as well as protecting of public health from counterfeiting, fraud and trafficking [26].

#### Labelling Requirements

- Any pack (excluding the packs being exported from UK) that is to be shipped within the UK from 01 January 2025, must possess a ‘UK Only’ statement and notify MHRA of packaging changes. The inclusion of ‘UK Only’ can be done either by stickering (until 30 June, 2025) or pre-printed on outer cartons [5].
- **Notification and Approval Process:** The updated mock-ups must comply with the MHRA’s Regulation 267 of the Human Medicines Regulations. MHRA has to be notified by any of the following submission routes by 31 December, 2024:
- Any regulatory opportunity: As part of another regulatory procedure (such as Type IA variation).
- Self—certification: Up to 25 licenses can be self-certified in bulk by submission of mock-ups of products.
- Self—certification without initial electronic Common Technical Document (eCTD) submissions: Self- certification process can be followed by the updating eCTD sequence. The sequence should be updated within 31 December, 2025 after initial self-certification [27].
- **Joint EU/UK Packs:** The Joint EU/UK packs cannot be released into the UK market from 01 January, 2025. However, existing packs in the market can be sold until their expiry date. Any content relevant to EU market must be removed from the cartons, and a common inventory can be maintained for leaflets and foil [5].

#### Removal of the EU Falsified Medicines Directive (FMD)

As of 01 January 2025, NI will come under the MHRA, so any packs that are coming into the UK will not require FMD features as it is a requirement of EMA. The safety regulations which are covered by UK law, such as inclusion of the batch number, expiration date and other packaging requirements, remain unmodified. However, inclusion of a 2D Barcode (which meets the global ISO/IEC standards) can still be continued if not uploaded to any European repository system. The anti-tamper packaging feature will be encouraged [5].

#### Supply Route—Green and Red Lane System

The Windsor Framework has created a streamlined UK internal market scheme, also known as the ‘green lane’, for goods remaining in the UK (trade between GB and NI). Goods that are at risk of entering EU single market (e.g.: Ireland) must pass through red lane, which includes comprehensive checks and controls. The new approach is based on data sharing, labelling of selected items and monitoring so that the single market in EU is protected and a uniform supply is maintained throughout the UK-wide [28].

#### The Northern Ireland MHRA Authorised Route (NIMAR)

The Windsor Framework will not have an impact on NIMAR’s ability to operate and support the importation of pharmaceuticals into NI. From 01 January 2025, upon commencement of the Windsor Framework, it is estimated that NIMAR will no longer be required to support the supply of medicines to NI as a Green Lane is established for the trade of goods between the UK and NI, However it is still effective in cases of any product shortage or unavailability of NI compliant pack [29].

#### Advertising and Promotion of Products

The advertising and promotion of products will be monitored by MHRA as per the Blue Guide across the entire UK.

Using the same advertisement for both UK and EU is advised to be avoided. However, the UK-audience should not be misled and the material has to be compliant with UK Summary of Product Characteristics (SPC) and should fulfill Part 14 of Human Medicines Regulations. The MHRA looks into any complaints such that no materials that are misleading, and those which fail to comply with UK’s legal requirements are withdrawn immediately. Complaints can be made as per the Blue Guide of MHRA [27, 30].

#### Pharmacovigilance

All MAHs, irrespective of license category, must send all UK-wide ICSRs and serious non-UK ICSRs that comply with the reporting requirements of MHRA from 01 January, 2025.

- **Category 1:** The PSURs of all Category 1 products are to be mandatorily submitted to MHRA. Unless the same product is authorized in EMA as well, it is not mandate to submit any ICSRs to EMA.

- Qualified Person for Pharmacovigilance (QPPV): It is mandatory to have a QPPV in UK, if he/she is established in the EU, then a National Contact Person (NCP) must be appointed in UK [31].
- Pharmacovigilance System Master File (PSMF): It must comply with MHRA and follow the UK submission metrics.

- **Category 2:** PSUR submission is not mandatory to MHRA, if available in EMA repository systems. However, ICSRs have to be submitted to MHRA.

- QPPV: A QPPV for both EU and UK are necessary. However, if a QPPV has already been appointed in the EU, MAH can appoint a National Contact Person (NCP) for UK.
- PSMF: For a product registered in MHRA, PSMF must comply with MHRA metrics primarily. However, in cases of ambiguity MAHs can also refer to EMA regulations [31].

#### Pediatric Requirement

The Pediatric Applications for medicines that transferred from the EU to UK on 01 January 2025, will also be affected if they are still under registration. Pediatric Investigational Plans (PIPs) are a mandatory requirement for new marketing authorisation applications in the UK. Any product whose PIP/waiver opinion is available at the EMA will be acknowledged by MHRA for the authorisation of products. However, variations are to be filed wherever applicable based on the UK’s PIP requirements. PIPs under Category 1 products will be governed by UK law, while those under Category 2 by UK law or EU law as applicable [22, 32].

#### Variation

The products authorised under CAP will not be applicable from 01 January, 2025, in NI. The variations pending in these products along with any transfer of MAs to MHRA will have to be applied again in MHRA as applicable. The pending variation in PLGB applications will be automatically applicable UK-wide. Previously, if the product was authorised by the EMA in NI, the goods required a variation to be filled in MHRA, to secure the Unfettered access route, which will no longer be required [33].

**Figure 1:**
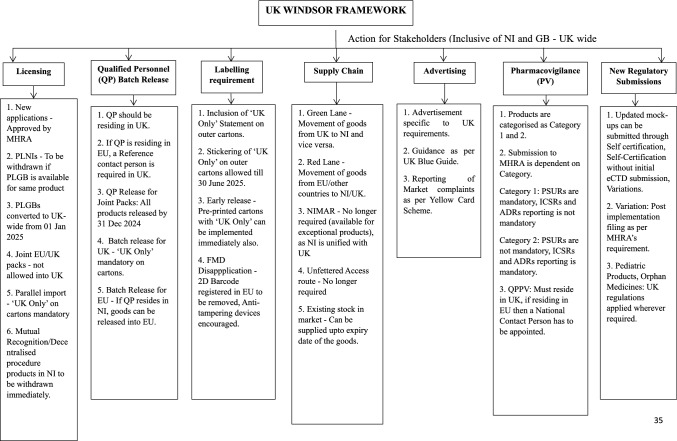
Action for Stakeholders on Windsor Framework Implementation.

The descriptive analysis of actions required from a Stakeholder to market in UK is provided in Fig. 1.

### The Evaluation of Windsor Framework

The implementation of Windsor Framework in NI is outlined using 2 aspects, Pre-implementation i.e., the scenario until 31 December, 2024 and the Post-implementation changes which has commenced from 01 January 2025, as referred in Table 1.

**Table 1:** Comparative Study of Windsor Framework Implementation in Northern Ireland (NI).

Sl. No	Parameters			Pre- implementation	Post- implementation
1.	Regulatory Authority for NI	EMA		✓	X
		MHRA		✓	✓
2.	Falsified Medicines Directive (FMD)/Serialisation	A. Unique Identifiers (2-D Barcode)		✓	X
		B. Anti-tampering device		✓	Not mandatory
3.	Labelling	A. UK—Only stickers on Packs		✓ (Printed/stickered)	✓ (Printed on Outer Packs)
		B. Stickering		✓	X
		C. Joint EU/UK cartons		✓	X
		D. Joint EU/UK Primary Packaging & Leaflets		✓	✓
4.	Regulatory Compliance	A. Dual Regulations—EMA/MHRA		✓	X
		B. UK-wide regulation		X	✓
5.	Licensing	A. PLGB/PLNI/EMA—Marketing Authorisation		✓	X
		B. UK-wide License		X	✓
6.	Supply route	A. NIMAR—Northern Ireland MHRA Authorised Route		✓	X
		B. Green Lane (Movement between GB and NI)		✓ (Detailed documentation)	✓ (Minimal checks)
		C. Red Lane (Movement across EEA and NI)		✓ (Minimal checks)	✓ (Detailed documentation)
7.	Advertising and Promotion	A. Directive 2001/83/EC		✓	X
		B. Blue Guide		✓ (Not mandatory)	✓ (Mandatory)
8.	Monitoring of Products	A. Drug precursor chemical control (Import–Export Licenses for trade between GB and NI)		✓ (Import–Export Licenses required from both GB/NI)	X
		B. Additional Monitoring of Medicines in NI		✓ (Both MHRA-EMA)	✓ (MHRA Only)
9.	Expedited Pathway for Registration of medicines in NI	PRIME—Priority Medicines		✓	X
		ILAP—Innovative Licensing and Access Pathway		X	✓
10.	Pharmacovigilance	Reporting of Periodic Safety Update Reports (PSURs)- Category 1	A. UK only licence—MHRA Reporting	✓	✓
			B. UK only license—EMA Reporting	✓	X
			C. UK and EU licence—MHRA Reporting	✓	✓
			D. UK and EU licence—EMA Reporting	✓	✓
		Reporting of Periodic Safety Update Reports (PSURs)- Category 2	A. UK and EU licence—MHRA Reporting	X	NA
			B. UK and EU licence—EMA Reporting	✓	✓

## Discussion

The Windsor Framework requirement arose due to the drawbacks of the Northern Ireland Protocol. Since the situation of Brexit is one of a kind, the implementation of the Windsor Framework needs to be critically reviewed. The consequences that might arise due to the shortcomings of the Framework and the possible hurdles that can arise during this transition period for an MAH or manufacturer are discussed as below:

### Consequences

#### In Northern Ireland

- **Role of the MHRA vs. EMA Post-Windsor Framework:** Under the Windsor Framework, the MHRA directly oversees pharmaceutical authorizations for Northern Ireland leaving a very limited role to the EMA in NI. Nevertheless, the EMA’s authorisation stays important for medicines making their way into the EU from Northern Ireland. For instance, there would be MHRA requirements for companies that do business in the UK specifically, and EU requirements for products gaining approval from EMA, making it slightly difficult for manufacturers outside Europe who are involved in both the EU and UK [34].
- **Supply Chain and Compliance in Customs for Importation of Medicines:** The provisions made under the Windsor Framework also affect customs compliance, in particular for the supply chain of pharmaceuticals. The Green Lane helps to keep compliance costs and trading times within the UK more efficient for the supply of medicines [35]. On the other hand, any product that is stored or manufactured in the NI and has to enter the EU has to pass through the red lane which is governed by the detailed EU customs. Pharmaceutical firms are therefore required to adhere to all the EU customs regulations, including those pertaining to electron customs declarations made under the EU Custom Data Model (EU CDM) which is a standardized framework for custom data management across the EU. To ensure compliance with EU pharmaceutical rules, medicine manufacturers must submit complete product details such as batch release certificates, GMP (Good Manufacturing Practice) compliance certifications, and safety data. Before a product may be sold in the EU, manufacturers must also adhere to import control procedures, which may involve regulatory verifications and inspections, to ensure that it complies with the EU’s Good Distribution Practices (GDP) and Falsified Medicines Directive (FMD). The chances of a drawback to occur in Windsor Framework lies in the Red Lane. Though products can move across the GB-NI sea border within minimal checks, a product moving across the shared border of Ireland and NI will face the strict scrutiny of EU. Hence, products which are sourced exclusively from NI/ GB will be reduced drastically [36–38].
- **Removal of EU FMD:** The falsified Medicines Directive is intended to dismantle entry of counterfeit medicines into the EU supply chain and to guarantee the quality of medicines which reach EU-based patients. The UK has had to seek other routes to sustain medicines safety in the country [39]. In NI, the EU FMD has been opinionated to be replaced by new measures by the UK including better GMP, transparent supply chain regulation through the MHRA and additional safety features. However, this might reduce the chances of duplication of regulation in NI. The disapplication of FMD features with only the strict monitor of manufacturing and supply cannot guarantee a consumer on the authenticity of a product [40].

#### In Great Britain (England, Scotland and Wales)

The Windsor Framework was implemented to safeguard the single market of the EU and to reinstate the place of Northern Ireland in the UK. Manufacturers or MAHs in Great Britain will also face the stress of the regulatory shift occurring around them, as outlined below:

- **GB-only Licensing Process:** Even if Marketing Authorisation Holders (MAHs) wish to market their products only in Great Britain, they are no longer be able to apply for a GB-only MA (a PLGB covering only Great Britain) after 31 December, 2024. After 01 January, 2025, the MHRA will no longer allow manufacturers to apply for GB-only MAs, and any exception will be assessed to issue GB-only MAs upon consideration to protect the patient’s health interest within the UK. All the PLGBs were changed into UK-wide licenses immediately from 01 January, 2025. If an MAH possessed an additional product license covering Northern Ireland (PLNI) they were to be surrendered by 31 December, 2024 to avoid any further administrative dilemma [23].
- **Disapplication of EU FMD:** The disapplication of FMD is already in place in GB, since Brexit. Starting from 01 January, 2025, the disapplication of FMD becomes mandatory for all packs entering the UK- (including PLPIs). Before then, FMD features were active only in NI.

However, there are two more countries where FMD has not been implemented in the EU i.e. Italy and Greece who trade products into GB. This is a result of the fact that Greece and Italy each have their two-part label verification mechanism in place. More specifically, in the Italian system, when the product is dispensed, the adhesive top layer sticker is taken off while the second layer is revealed on exposure to UV light in Greece marking their unique nature of identifying a counterfeit medicine which will not have such barcodes. After FMD is completely removed from 01 January 2025, the license holder importing the goods into the UK is responsible for identifying any such fake, stolen or recalled stocks. Any counterfeit medications found throughout this process should be reported via the MHRA’s Yellow Card reporting system [41].

#### In the Republic of Ireland

Due to the traditionally established routes for medicines supply between Ireland and Northern Ireland, the implementation of the Windsor Framework will affect the MAHs in the EU; more specifically the Republic of Ireland due to the disapplication of the FMD features for NI. Medical repositories like wholesale distributors, hospitals or pharmacies will disconnect from the FMD system of the EU, mainly related to the UK packs that move into the EU markets. The UK continues to be the primary supplier of Exempt Medicinal Products (EMPs), also known as Unlicensed Medicines (ULMs), particularly for the medicinal products that do not have marketing authorisation (MA) in Ireland. If equivalent licensed products are unavailable in Ireland, these EMPs can be sourced from Northern Ireland or the UK market. However, once imported, they cannot be exported from the Irish market due to regulatory frameworks that strictly restrict their redistribution [42]. One such example is an anti-convulsant medicine Zonisamide oral suspension, which is prescribed to treat epilepsy, particularly as an adjunct therapy in partial seizures. In many markets, Zonisamide is available in capsules, the oral suspension formulation is not authorised in some countries including Ireland. Under such circumstances, where no authorised equivalent is available, Irish healthcare practitioners can procure it as an Exempt Medicinal Product from the UK or NI. This ensures that patients who require the medicine for the management of the disease can still get access despite its lack of marketing authorisation (MA) in Ireland. The regulatory measures, as outlined in the Windsor Framework, mandate that these imported medicines (i.e. EMPs) must remain within Ireland and cannot be redistributed to other markets that play a critical role in ensuring an uninterrupted medicine supply to Ireland. The framework provides a structural approach to maintaining regulatory alignment and pharmaceutical supply chain integrity, safeguarding patient access to essential medicines [42, 43].

Presently, the Irish Medicines Verification System (IMVS) can be used to identify the data of these packs from the UK, hence, any dispensaries will not receive alerts on the products. Post implementation of the Windsor Framework, these packs will generate an alert on the IMVS when the 2D Barcode is scanned as this data will be removed from the UK system along with the disapplication of FMD. The Irish Medicines Verification Organization (IMVO) has been closely working with the Department of Health, Health Products Regulatory Authority (HPRA) and other government authorities in the Republic of Ireland to minimize the effects of new Framework implementation (disapplication of FMD) in the Northern Ireland [44].

## Evaluation of UK MHRA-Windsor Framework VS EU Regulations in NI

Since the Brexit, Northern Ireland has faced concerns regarding the supplies for about 80% of its medicines which were sourced from GB. The necessity of the Windsor Framework can be understood by evaluating the landscape of Northern Ireland, Great Britain and the Republic of Ireland, in terms of medicines supply. Though there are many advantages to the implementation of the Windsor Framework as it is evaluated the proper implementation of the regulations can solve almost all problems faced by NI public in the sector of Pharmaceutics [45]. The implementation itself is a work in progress and needs to be compared with the EU regulations to quantify the possible success rate of this transition. The evaluation of the Windsor Framework with that of EU regulation is dynamic and cannot be restricted to a set number of parameters. However, based on the current scenario of the UK-EU market and available data, the comparative evaluation that can be expected upon implementation of the UK Windsor Framework with that of EMA regulations has been tabulated below in Table 2.

**Table 2:** UK-Windsor Framework vs. EU Regulations.

Comparative analysis	UK Windsor framework	EU pharmaceutical regulations (EMA)
Regulatory Authority	MHRA governs the regulation of medicinal products in UK including Northern Ireland (NI) It operates independently of EMA but maintains cooperation wherever applicable	European Medicines Agency (EMA) oversees regulations of medicinal product across its member states
Marketing Authorisation	All product licenses will be primarily considered to be UK wide All UK application must go through MHRA-specific approval process post Windsor Framework No automatic cross recognition with EMA, separate approval process required for products already authorised in EU	Multiple pathways: Centralised, Decentralised, Mutual recognition and National procedure remains unchanged for market approval across all member states of EU
Pharmacovigilance	Independent Pharmacovigilance system is followed by MHRA Yellow card scheme for reporting adverse event reporting (ADRs)	EudraVigilance database for monitoring and reporting adverse event reporting (ADRs) across EU member states
Pricing and Reimbursement	MHRA determines cost and reimbursement of medicines Separate pricing policies are available for entire UK	Each EU member state determines pricing and reimbursement though EMA encourages transparency and cross border referencing of prices
Labelling	Windsor establishes new labeling (‘UK Only’) requirements in accordance with MHRA	Labelling requirements are established as per EMA directive
Regulatory Compliance	MHRA maintains a uniform compliance in Good Manufacturing Practice (GMP) inspections and safety reporting	EMA coordinates with all member states to ensure organized approach of regulation
Market Access and Trade	Access to any authorised product is uniform throughout UK (including NI) Trade barriers cease to exist within the UK as facilitated in Windsor Framework	Market access in EU single market is independent of authorisation by MHRA Trade barriers will come into existence only between EU and NI

## Conclusion

Comparing the Windsor Framework to the EU regulatory framework for medicines demonstrates that there are differences that exist in terms of medicines approval procedures, requirements for compliance and commercialization. Analysis of primary and secondary sources depicts that, although simplification of procedures is the evident goal of the new Windsor Framework with the unification of NI into the UK, there are layers of complexity that have been brought for medicines manufacturers due to changing regulations in Great Britain, Northern Ireland.

Any regulatory modifications will have significant consequences for pharmaceutical firms. It remains clear that structural differences between the new UK framework and EU structure could impact public health through the potential extension of the time that medicines may take to come into the market. It is possible to see changes in international trade relations because companies will have to adjust their positions by these new regulations.

As reviewed in this study, the Windsor Framework has many provisions for the MAHs to assist and promote the integral trade changes occurring in Northern Ireland. Hence, an MAH who wishes to provide an uninterrupted supply of their products in the UK has to keenly assess the provisions of the Windsor Framework before implementing it upon their market supply.

Some possible limitations of this study include the dynamic nature of the regulations and their possible biases in the available literature. Hence, there is a need to periodically update the review and analyze the regulatory environment relative to the changing nature of the pharmaceutical industry.

## Data Availability

No datasets were generated or analysed during the current study.
